# Navigating Pediatric Atypical Hemolytic Uremic Syndrome: A Two-Year Case Series From Eastern India

**DOI:** 10.7759/cureus.95314

**Published:** 2025-10-24

**Authors:** Swarnim Swarnim, Megha Saigal, Priyanka Priyanka, Yagavan P, Nutan Sharma, Harleen Kaur

**Affiliations:** 1 Pediatrics, All India Institute of Medical Sciences, Patna, Patna, IND; 2 Nephrology, All India Institute of Medical Sciences, Patna, Patna, IND

**Keywords:** atypical hemolytic uremic syndrome, clinical outcomes, complement system, kidney disease, thrombotic microangiopathies

## Abstract

Background

Atypical hemolytic uremic syndrome (aHUS) is an ultra-rare form of thrombotic microangiopathy that results from complement system activation. It represents a significant etiology of acute kidney injury among children. In India, aHUS is predominantly associated with anti-factor H antibodies, presenting unique diagnostic and therapeutic challenges. This study aims to delineate the clinical characteristics, immunological profile, management strategies, and outcomes of pediatric aHUS cases from a tertiary center in Eastern India.

Methodology

We conducted a retrospective, observational, case series at a tertiary care hospital in Eastern India, including seven pediatric patients diagnosed with aHUS between January 2023 and December 2024. Diagnosis was based on a clinical triad (acute kidney injury, microangiopathic anemia, thrombocytopenia), exclusion of Shiga toxin-associated cases and secondary causes, confirmation with complement/anti-factor H antibody testing, and, where feasible, genetic analysis. Patients received plasma exchange, immunomodulators, and supportive care. Data were analyzed using descriptive statistics.

Results

A total of seven children (median age = 7 years, six females) were treated for aHUS. *CFHR1-CFHR3* deletions and anti-factor H antibodies were each identified in 43% of patients. Plasma exchange and steroids formed the therapeutic mainstay. Hematological remission was achieved in 71% of cases within one week, and 43% attained full renal recovery. However, 29% progressed to chronic kidney disease or remained dialysis-dependent, and the remaining 29% showed only limited renal improvement. Early initiation of plasmapheresis and immunosuppression was associated with better renal outcomes, while lack of access to eculizumab and genetic testing remained significant barriers.

Conclusions

Pediatric aHUS in Eastern India demonstrates a high burden of anti-factor H antibody-mediated disease and genetic complement abnormalities. Early plasmapheresis and immunomodulator use result in improved hematological and renal outcomes. There is a critical need for enhanced access to complement inhibitors and diagnostic tools to optimize management and prognosis in resource-constrained settings.

## Introduction

Hemolytic uremic syndrome (HUS), a leading cause of acute kidney injury in children, presents significant clinical challenges, particularly in its atypical form (aHUS). A significant proportion of patients may require renal replacement therapy, and approximately one-third of patients exhibit features of chronic kidney disease [1]. Most HUS cases globally follow gastrointestinal infections, such as those caused by Shiga toxin-producing *Escherichia coli*. aHUS, however, arises from dysregulated complement pathways either from genetic mutations in genes of complement components or regulatory proteins of the alternate complement pathway, such as complement factor B, C3, complement factor H (CFH), complement factor I, and membrane cofactor protein, or is anti-FH (aFH) antibody-mediated. This distinction is crucial in regions like India, where improving standards of hygiene and healthcare evolution have altered HUS epidemiology, notably reducing *Shigella*-associated cases [2,3]. Studies have found that aFH antibody-associated aHUS comprises about 5-25% of cases of aHUS in European and North American cohorts, whereas it is seen in ~50% of patients in India [1,4]. Understanding the local disease profile, genetic variants, therapeutic interventions, and outcomes in Indian children with aHUS is crucial for optimizing management strategies, planning resource allocation, and informing public health policy. However, there is a paucity of systematically collected data addressing these aspects within the Indian pediatric population.

This study addresses these gaps by presenting a two-year series of pediatric aHUS cases from a tertiary care center in Eastern India. The primary aim of this study was to characterize the clinical and genetic spectrum of pediatric aHUS in a resource-limited Indian setting. The secondary aim was to evaluate the response to plasmapheresis-based therapy in this cohort. Through this study, we have tried to inform both clinical practice and policy in regions where access to targeted therapies remains limited.

## Materials and methods

Study design and setting

This retrospective, observational case series was conducted in the Departments of Pediatrics and Nephrology at the All India Institute of Medical Sciences (AIIMS), Patna, a tertiary referral center in Eastern India. Medical records were reviewed for children admitted with a diagnosis of aHUS between January 2023 and December 2024.

Patient selection

Children under 18 years of age presenting with clinical and laboratory evidence of thrombotic microangiopathy, including microangiopathic hemolytic anemia, thrombocytopenia, and acute kidney injury, were included if aHUS was diagnosed by exclusion of Shiga toxin-associated (typical) HUS and other secondary causes (infectious, malignant, autoimmune, or drug-induced etiology). Cases with incomplete records or alternative diagnoses were excluded.

Diagnostic workup

Assessment involved complete blood count, peripheral blood smear for schistocytes, lactate dehydrogenase (LDH), reticulocyte count, renal and liver function tests, and urinalysis. Complement levels (C3/C4), direct Coombs test, and autoimmune profiles (anti-nuclear antibody (ANA), vasculitis panel) were performed when indicated. Anti-FH antibody testing was conducted in selected patients. Genetic diagnosis (whole-exome sequencing or multiplex ligation-dependent probe amplification (MPLA) for *CFHR1/CFHR3*) was performed where feasible. Renal biopsy was performed for diagnostic clarification when required. Screening for infections (malaria, dengue, kala-azar, leptospirosis) was performed in all cases.

Data collection

Demographic details, presenting complaints, laboratory parameters, treatment modalities (plasma therapy, immunosuppression, dialysis), and patient outcomes were systematically extracted and tabulated. Laboratory investigations were documented with reference ranges, and genetic findings were classified according to the American College of Medical Genetics (ACMG) guidelines.

Statistical analysis

Data were analyzed using SPSS version 27 (IBM Corp., Armonk, NY, USA). Continuous variables are reported as medians and interquartile ranges, and categorical variables are reported as counts and percentages. Given the small cohort, analyses were descriptive only.

Ethical approval

The study protocol was approved by the Institutional Ethics Committee, All India Institute of Medical Sciences, Patna (approval number: AIIMSPat2025IEC1445). Written informed consent for participation and publication was obtained from parents or guardians for all patients. Data were anonymized and handled in compliance with institutional policies.

## Results

The cohort comprised seven children, with a median age of seven years (range = 3 to 16 years), including six females and one male. All patients were diagnosed with HUS based on standardized criteria (acute kidney injury, microangiopathic anemia, thrombocytopenia). Additional complaints included swelling, hematuria, jaundice, abdominal pain, and systemic symptoms. Genetic testing identified *CFHR1/CFHR3* deletions in three (43%) patients, a *CFH* missense variant in one patient, and anti-FH antibodies were detected in three (43%) patients. Plasma-based therapy and immunosuppression were administered to all. Hematological recovery occurred in five (71%) patients within three to seven days of treatment. Renal outcomes varied: three (43%) patients achieved complete renal recovery by day 7-10, while two (29%) progressed to chronic kidney disease or required ongoing dialysis, and two (29%) showed limited renal recovery.

Patient details

Patient 1

A 16-year-old girl presented with symptoms including vomiting, blood in urine, and jaundice over four days. She experienced non-projectile vomiting and had yellowish eye discoloration without any abdominal pain. Laboratory results indicated severe anemia, a low platelet count, deranged kidney function test (urea = 118 mg/dL, creatinine levels = 3.61 mg/dL), elevated bilirubin levels, with a significant presence of schistocytes (>5%) in her peripheral blood smear, indicative of hemolysis. The direct Coombs test was negative. Dengue, malaria, kala azar, and leptospirosis were negative. C3 levels were reduced, and C4 was normal. The ANA screening profile and vasculitis profile were negative. Antibodies against complement H were negative (Table 1).

**Table 1:** Laboratory investigations of all patients at presentation. LDH = lactate dehydrogenase; FH = factor H

Parameter	Case 1	Case 2	Case 3	Case 4	Case 5	Case 6	Case 7	Reference range
Hemoglobin (g/dL)	6.5	6.8	4.7	6.2	9.4	3.6	4.6	11–14
Platelets (/mm³)	35,000	40,000	75,000	10,000	35,000	29,000	55,000	150,000–450,000
Schistocytes (%)	>5	10	6	12	5	5.6	10	<1
Urea (mg/dL)	118	395	113.3	200.6	294	87	84.3	7–20
Creatinine (mg/dL)	3.61	2.95	1.55	8.16	6.26	1.01	2.18	0.5–1.2
LDH (U/L)	1,100	2,245	1,650	1,627	>5,000	7,399	2,568	<450
C3 (g/L)	Low	Low	Low	Normal	Low	Low	Low	0.9–1.8
Anti-FH antibody (AU/mL)	Negative	>2,400	Negative	>2,400	2,028	1,853	>2,400	Negative

A targeted exome sequencing revealed a large homozygous deletion (~57.11 KB) on chromosome 1 (chr1:g.(?_196774887)_(196831999_?)del) (GRCh38), encompassing the *CFHR1* and *CFHR3* genes. Deletions encompassing these genes have been reported in aHUS [5]. The deletion was classified as a variant of uncertain significance as per ACMG guidelines. This large deletion was not further delineated/confirmed with MLPA/microarray, as it was consistent with clinical findings, as well as due to financial constraints.

The patient was managed for atypical HUS with plasma exchange and pulse methylprednisolone. Her condition improved with treatment, showing hematological remission and renal function recovery within three and seven days, respectively (Table 2).

**Table 2:** Clinical profile and treatment outcomes of all pediatric atypical hemolytic uremic syndrome cases. PLEX = plasma exchange; FH = factor H; TMA = thrombotic microangiopathy; CKD = chronic kidney disease; HUS = hemolytic uremic syndrome

Patient ID	Age/Sex	Presenting complaints	Genetic/Antibody findings	Renal biopsy	Treatment	Hematological recovery (days)	Renal outcome
Patient 1	16/F	Vomiting, hematuria, jaundice	*CFHR1*/*CFHR3* homozygous deletion	Not done	PLEX, steroids	3	Full recovery in 7 days
Patient 2	12/F	Swelling, hematuria, oliguria	Anti-FH antibody positive	Suggestive of TMA	Plasma infusion, steroids, cyclophosphamide	5	CKD, on dialysis
Patient 3	4/M	Nephrotic syndrome, fever, sepsis	*CFH* missense variant (c.157C>T)	Suggestive of TMA	PLEX, dialysis, immunosuppression	12	Dialysis-dependent
Patient 4	4/F	Abdominal pain, dyspnea	Anti-FH antibody positive	Not done	PLEX, steroids, dialysis, ventilation	7	Limited recovery
Patient 5	7/F	Swelling, jaundice, oliguria	*CFHR1*/*CFHR3* heterozygous deletion, Anti-FH positive	Suggestive of HUS with focal tubular necrosis	PLEX, steroids, dialysis, ventilation	3	Recovery in 10 days
Patient 6	3/F	Vomiting, paleness of the body	Homozygous deletion: CFHR3 (upstream region, exons 1,2,3,4, 6, and intron 4), CFHR1 (introns 1, 3, and exons 2, 4, 5, 6)	Not done	Hemodialysis, plasma therapy, steroids, mycophenolate mofetil	7	Recovery in 7 days
Patient 7	10/F	Generalized swelling, paleness of the body, headaches	Not done	Not done	Plasma therapy, steroids, mycophenolate mofetil, antihypertensives	5	Limited recovery

Patient 2

**Figure 1:**
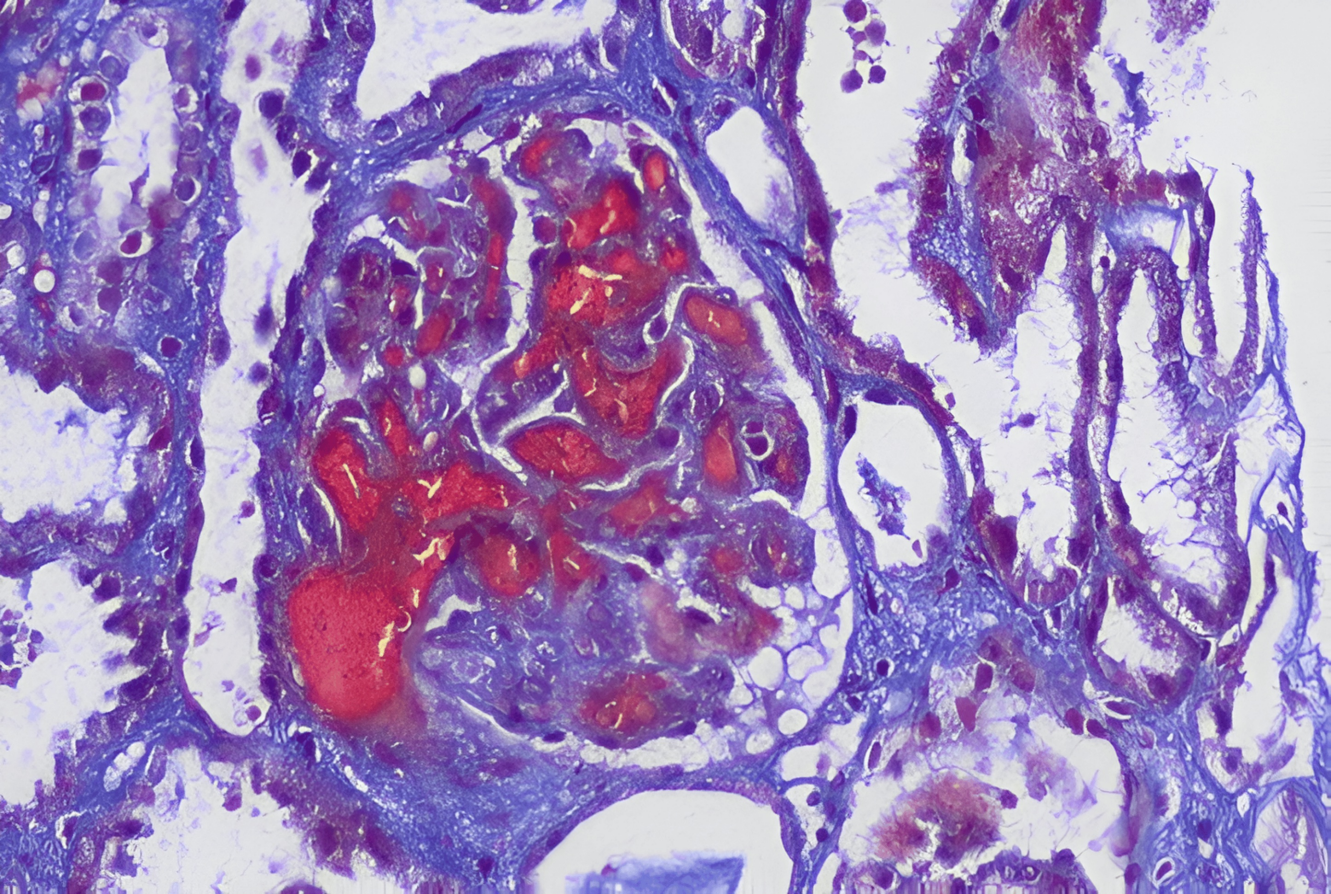
Renal biopsy of Patient 2: glomerular capillary lumina with fibrin thrombi and fluffy-appearing mesangial areas (masson trichrome, 200×).

**Figure 2:**
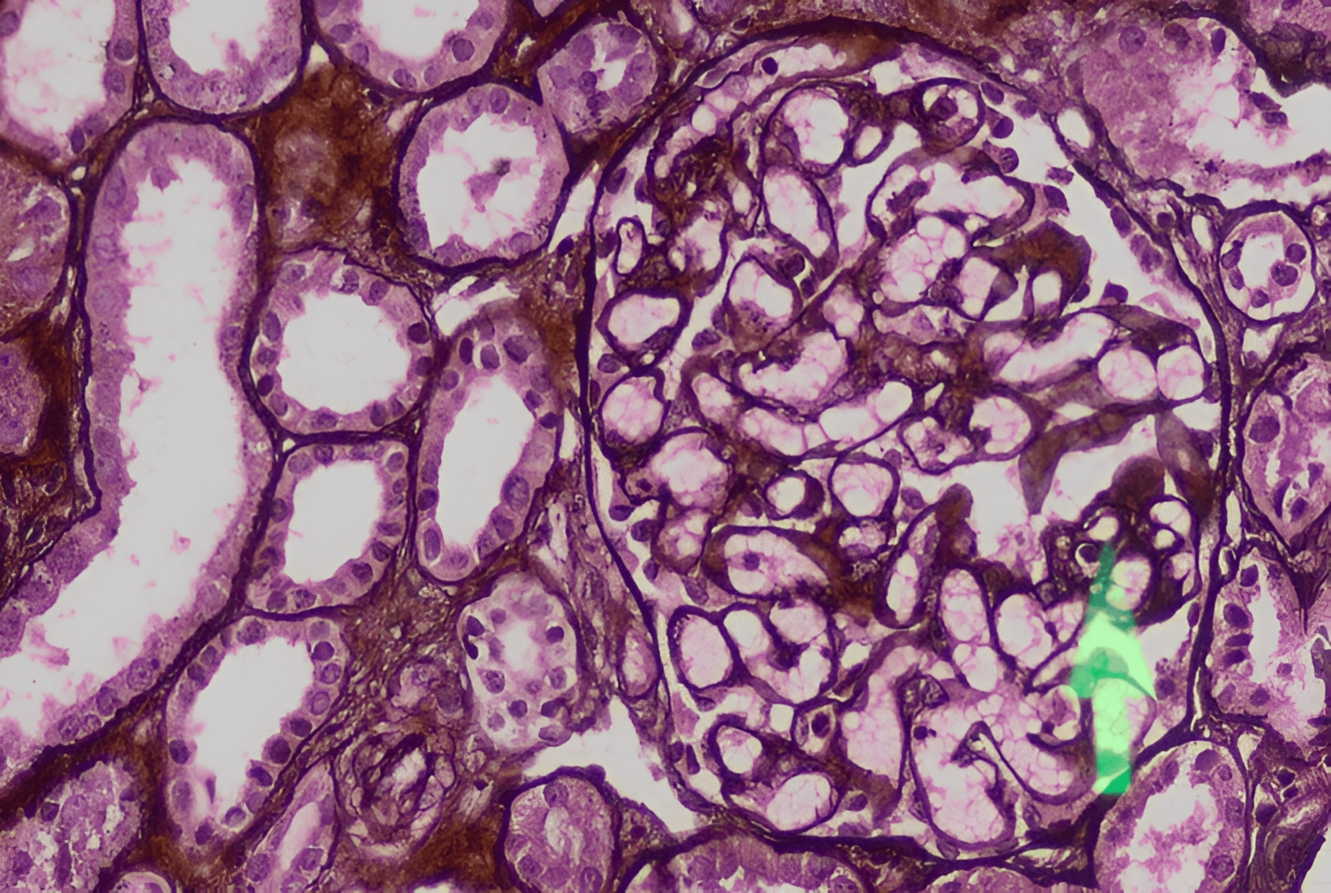
Renal biopsy of Patient 2: Focal reduplication of the glomerular capillary basement membrane (arrow) (silver, 200×).

A 12-year-old girl had symptoms of body swelling, paleness, hematuria, and reduced urine output for about a week. She also experienced episodes of vomiting. On examination, she was conscious, with elevated blood pressure, pallor, icterus, and edema, but no organomegaly or lymphadenopathy was noted. Laboratory tests revealed severe anemia, thrombocytopenia (platelet count = 40 × 10^9^/L), and kidney dysfunction (urea = 395 mg/dL, creatinine = 2.95 mg/dL), with 10% schistocytes on the peripheral smear and elevated LDH levels (Table 1). Renal biopsy confirmed features of thrombotic microangiopathy. Histopathology demonstrated duplication of the glomerular basement membrane with mesangiolysis and endothelial swelling. Capillary loops showed a “tram-track” appearance on silver stain, consistent with endothelial injury (Figures 1, 2).

She had a high anti-FH antibody level, suggesting aHUS. Financial constraints limited her genetic testing and treatment options. She received plasma infusion therapy and immunosuppression with methylprednisolone and cyclophosphamide. Hematological response was observed in five days, but her renal function did not improve with progression to chronic kidney disease requiring maintenance dialysis (Table 2).

Patient 3

A four-year-old boy was being treated by a private practitioner with a history of steroid-resistant nephrotic syndrome with tacrolimus and had received two doses of rituximab one month back. He presented with fever, multiple pustules on the face, facial swelling, and paleness. His condition worsened with sepsis and acute kidney injury. Laboratory tests revealed anemia, thrombocytopenia, deranged kidney function, significantly elevated schistocytes (6%) and reticulocyte counts (10.5%), and negative ANA. C3 was low, and C4 was normal (Table 1). A renal biopsy confirmed thrombotic microangiopathy. The child also had low IgG for age, for which he received intravenous immunoglobulin. He had negative anti-FH antibodies. However, whole-exome sequencing revealed homozygous missense variants (chr1:g.196673076C>T, c.157C>T,p. Arg53Cys) in the *CFH* (+) gene (GRCh38) (Table 3).

**Table 3:** Genetic findings of Patient 3: whole-exome sequencing identified a homozygous missense variant in CFH (c.157C>T; p.Arg53Cys), previously reported in association with atypical HUS. HUS = hemolytic uremic syndrome

Gene (transcript)	Location	Variant	Zygosity	Disease/OMIM	Inheritance	Classification
CFH (ENST00000630130.2)	Exon 2	c.157C>T (p.Arg53Cys)	Homozygous	Complement factor H deficiency (OMIM#609814)/Atypical hemolytic uremic syndrome-1 (OMIM#235400)	Autosomal recessive	Likely pathogenic (PS1, PM1, PM2, PP3)

This variant has been previously reported in aHUS and was classified as likely pathogenic according to ACMG guidelines [6]. He received plasmapheresis followed by plasma infusions and dialysis. Hematological remission was achieved in 12 days, but acute kidney injury persisted, and he continued on maintenance hemodialysis before the parents took him home against medical advice because of financial constraints (Table 2).

Patient 4

A four-year-old girl presented with a two-month history of abdominal pain, intermittent vomiting, periorbital puffiness, and abdominal swelling. In the last week, she experienced severe dyspnea and was unable to lie flat due to respiratory distress. Upon arrival, she showed signs of respiratory failure from pulmonary edema and cardiac failure, requiring intubation and mechanical ventilation. She was treated with inotrope milrinone and supportive care, and started on peritoneal dialysis for hemodynamic instability. Laboratory tests indicated hemolytic anemia, thrombocytopenia, elevated LDH, and significant schistocytes (12%), but normal ANA and complement levels (Table 1). As she had high anti-FH autoantibody (>2,400 AU/mL), she was started on plasmapheresis and methylprednisolone pulse therapy. She showed gradual improvement with hematological remission; however, her acute kidney injury showed limited recovery (Table 2).

Patient 5

**Figure 3:**
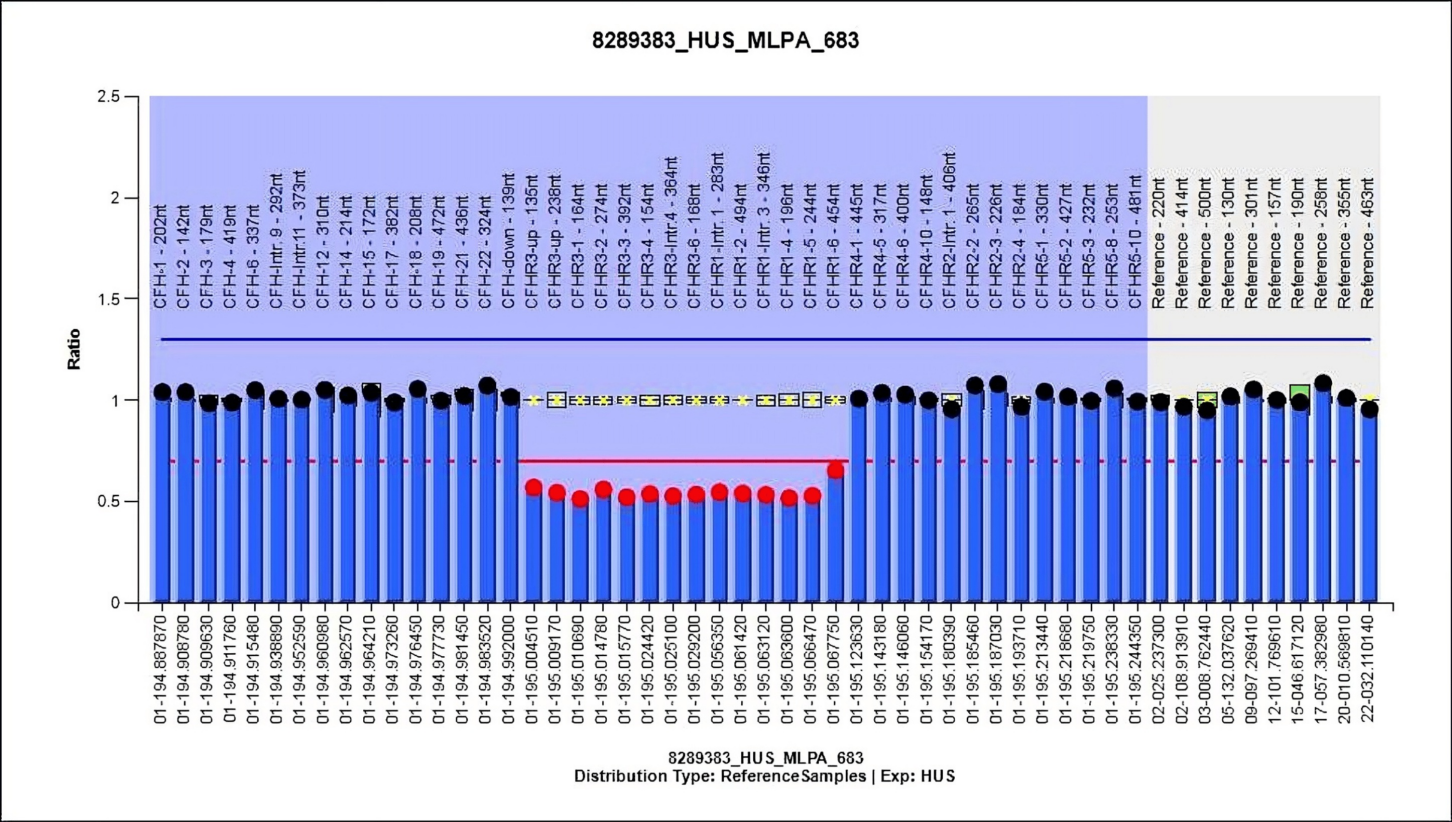
Genetic analysis of Patient 5: MLPA-based copy number variation analysis demonstrated homozygous deletion of CFHR3 and CFHR1, a well-recognized susceptibility factor for anti-factor H antibody-associated atypical HUS. MPLA = multiplex ligation-dependent probe amplification; HUS = hemolytic uremic syndrome

A seven-year-old girl exhibited generalized body swelling, yellowish discoloration of the sclera and urine, altered sensation, and reduced urine output. Upon admission, she was in respiratory failure due to pulmonary edema and cardiogenic shock. She required intubation, mechanical ventilation, and immediate hemodialysis. Laboratory investigations showed hemolytic anemia, elevated liver enzymes, and renal impairment (Table 1). A renal biopsy indicated HUS with focal tubular necrosis. A high anti-FH level of 2,028 AU/mL was noted. Genetic analysis revealed a heterozygous deletion of the region encompassing the upstream region, exons 1, 2, 3, 4, 6, and intron 4 of the *CFHR3* gene and introns 1, 3, and exons 2, 4, 5, 6 of the *CFHR1* gene (Figure 3).

Following treatment with hemodialysis, plasma infusion, steroids, and mycophenolate mofetil, she achieved hematological remission in three days and showed significant improvement in kidney function within 10 days (Table 2).

Patient 6

**Figure 4:**
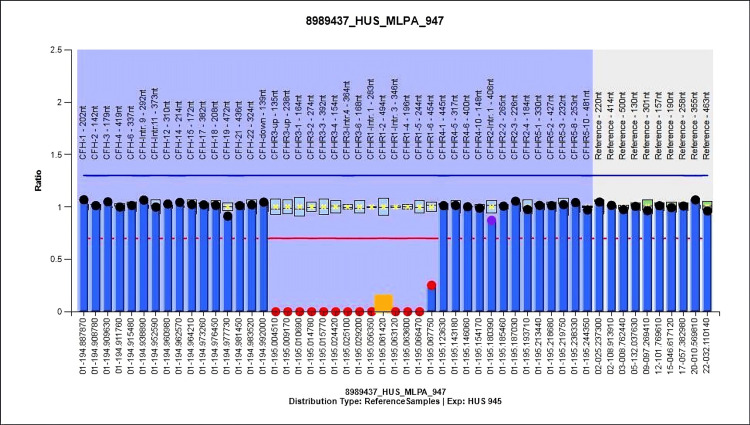
Genetic analysis of Patient 6: MLPA ratio chart showing heterozygous deletion involving CFHR3 (upstream region, exons 1-4, 6, intron 4) and CFHR1 (introns 1, 3, exons 2, 4-6). MPLA = multiplex ligation-dependent probe amplification

A three-year-old female presented with vomiting and paleness of the body for 10 days. She experienced non-projectile vomiting. There was no history of jaundice, fever, loose stools, or altered sensorium. Laboratory results indicated severe anemia, a low platelet count, and deranged kidney function test (creatinine levels = 1.01 mg/dL) with a significant presence of schistocytes (>5%) in her peripheral blood smear, indicative of hemolysis. The direct Coombs test was negative. Dengue, malaria, kala azar, and leptospirosis were negative. C3 levels were low (Table 1). The ANA screening profile was negative. A high Anti-FH level of 1,853 AU/mL was noted. Genetic analysis revealed a homozygous deletion of the region encompassing the upstream region, exons 1, 2, 3, 4, 6, and intron 4 of the *CFHR3* gene and introns 1, 3, and exons 2, 4, 5, 6 of the *CFHR1* gene (Figure 4).

The parents had been informed of plasma exchange, but they refused due to financial constraints. The child received plasma therapy and started on an immunosuppressant steroid and mycophenolate mofetil. Hematological and renal remission occurred in seven days (Table 2).

Patient 7

A 10-year-old female presented with symptoms of generalized body swelling and paleness of body for 15 days. She also complained of headaches, severe grade, generalized, associated with blurring of vision, dizziness, and vomiting. There was no history of jaundice, loose stool, seizure, or altered sensorium. Laboratory tests revealed severe anemia, thrombocytopenia (platelet count = 55 × 10^9^/L), and kidney dysfunction (urea = 84.38 mg/dL, creatinine = 2.18 mg/dL), with 10% schistocytes on the peripheral smear and elevated LDH levels. She had a high anti-FH antibody level, suggestive of aHUS (Table 1). Financial constraints limited her genetic testing and treatment options. She received plasma infusion therapy and immunosuppression with steroids and mycophenolate mofetil. Hematological response was observed in five days, but her renal function showed partial improvement only (Table 2). Parents took her home against medical advice because of financial constraints.

## Discussion

The non-diarrhea-associated form of HUS, also known as aHUS, is not as rare as previously considered. While Shiga toxin-associated HUS is the chief form of the disease worldwide, our study confirms the ambiguity regarding the burden of this illness in India, highlighting the epidemiological differences in the Indian context. Consistent with the findings of the Indian Society of Pediatric Nephrology, our study identified a significant prevalence of anti-FH antibody-associated aHUS among Indian children. The age range was wide, spanning from three to 16 years, indicating that aHUS should be suspected not just in young children but also in adolescents presenting with thrombocytopenia, hemolytic anemia, and acute kidney injury.

Recent studies have emphasized the role of mutations in genes coding for complement regulators in the pathogenesis of aHUS. These mutations lead to defective regulation of complement activation [5,6]. Our findings on genetic mutations mirror global research, underscoring the role of complement and coagulation pathways in aHUS [7,8]. This study highlights the role of genetic analysis in definitively diagnosing aHUS, including in patients who had high anti-FH levels. As reported by several studies, anti-FH-associated HUS is strongly associated with deletions of the factor H-related 3 and 1 genes [9,10]. Hofer et al. reported *CFH* antibodies in 82% of pediatric patients with aHUS with homozygous *CFHR1* gene deletions and in 6% of patients without this deletion [11]. On the other hand, Sinha et al. found homozygous deletion of both *CFHR1* and *CFHR3* genes in 82.4% of patients having anti-CFH antibodies [12]. We could perform a genetic test in only two out of five patients with anti-FH-associated HUS, where one had a heterozygous deletion and another had a homozygous deletion in *CFHR3* (upstream region, exons 1, 2, 3, 4, 6, and intron 4), and *CFHR1* (introns 1, 3, and exons 2, 4, 5, 6). The previous child also had a significant family history of death of her father at 42 years of age due to acute kidney injury; however, with no known kidney disease in the past, the exact cause remains unknown.

While plasmapheresis and immunomodulators remain primary treatments in our setting, globally, the use of eculizumab, a C5 monoclonal antibody, has revolutionized aHUS management by directly targeting terminal complement activation [13]. Besides eculizumab, various new therapeutic alternatives with distinct action mechanisms tailored to specific mutation‐based pathophysiologies are under various stages of development [12,14]. However, eculizumab and ravalizumab are expensive drugs and not easily available in India. The reliance on plasmapheresis and immunosuppression in our study due to the unavailability of eculizumab is consistent with Indian guidelines [1]. These approaches, particularly plasmapheresis, are crucial for managing patients with suspected aHUS and significantly beneficial for those with anti-FH antibody-associated HUS. We found that early diagnosis and prompt initiation of plasma exchange were associated with improved renal outcomes in patients with aHUS, while lack of access to treatment due to financial constraints was tied to poor prognosis. Specifically, the cases highlighted those patients who received plasma exchange within one to two weeks of symptom onset showed higher chances of recovery of renal function. Our findings mirror those of Jain et al., who also reported high recovery rates with plasma exchange in a case series from India [15].

Collectively, these studies support the idea that where diagnostic capabilities are limited and access to eculizumab is restricted, plasma exchange should be promptly initiated in patients presenting with suspected thrombotic microangiopathy and severe renal dysfunction to improve the likelihood of renal recovery. The high cost and restricted availability of eculizumab in India present major therapeutic barriers for aHUS management. Cost-effectiveness analyses and national policy initiatives are urgently needed to expand access to complement inhibitors and establish affordable, regionally tailored treatment protocols. Collaborations for pooled procurement and increased funding for rare disease management could greatly improve patient outcomes in resource-limited settings.

This study provides one of the few systematically collected case series of pediatric aHUS from a resource-limited eastern Indian setting, integrating clinical, genetic, and immunological data with outcomes. Comprehensive exclusion of secondary thrombotic microangiopathy and standardized laboratory workflows support diagnostic accuracy.

This study has a few limitations. The small sample size, incomplete genetic testing in all patients, lack of long-term outcome data, and possible selection bias limit generalizability and causal inference. Limited therapeutic options reflected local resource constraints. Further prospective research is needed on larger samples of aHUS patients in this setting to better understand long-term prognosis and optimize management in the absence of eculizumab. Improved access to genetic and antibody testing would also help confirm diagnoses.

## Conclusions

In our series of seven pediatric aHUS patients, 43% harbored *CFHR1/CFHR3* deletions, and a similar proportion had anti-FH antibodies. Hematological remission was achieved in 71% of patients following plasma-based therapies, but only 43% showed full renal recovery, with 29% progressing to chronic kidney disease or remaining dialysis-dependent. Restricted access to eculizumab and limitations of genetic testing remain key challenges. These findings underscore the critical need for early diagnosis and plasma exchange in resource-limited settings and highlight the importance of local policy action to bridge therapeutic gaps.
